# Identification of key aromatic compounds responsible for sweetening sensory effect in Jinmudan black tea though flavoromics and molecular modeling

**DOI:** 10.1016/j.fochx.2025.103314

**Published:** 2025-11-29

**Authors:** Wu Yang, Wu Qingyang, Zhou Ziwei, Deng Tingting, Sun Yun

**Affiliations:** a College of Horticulture, Key Laboratory of Ministry of Education for Genetics, Breeding and Multiple Utilization of Crops, Fujian Agriculture and Forestry University, 15 Shangxiadian Road, Fuzhou 350002, PR China; b College of Materials Engineering, Fujian Agriculture and Forestry University, 63 Xiyuangong Road, Fuzhou 350002, China; c College of Life Science, Ninde Normal University, 1 Xueyuan Road, Ninde 352100, China; d Fujian Higher Education Research Center for Local Biological Resources, 1 Xueyuan Road, Ninde 352100, Fujian, China

**Keywords:** Innovative black tea, Sweetening sensory effect, Flavoromics, Molecular modeling

## Abstract

Innovative Jinmudan black tea shows pronounced sweet aroma and taste, but the mechanisms by which volatile compounds enhance sweetness remain unclear. This study combined flavoromics, molecular modeling, and sensory analysis to identify key aroma compounds responsible for the sweetening sensory effect. HS-SPME-GC–MS and multivariate analysis characterized 93 sweet-related volatiles, of which 30 were screened by Partial Least Squares-Discriminant Analysis (PLS-DA). Six representative compounds covering major sweet-aroma subtypes were selected for *in silico* analysis. Molecular docking and dynamics revealed stronger and more stable binding of these volatiles to the sweet receptor T1R3 than to T1R2. Aroma addition tests using a low-sweetness Jinmudan black tea confirmed that five compounds enhanced perceived sweetness, with (*Z*)-3-hexen-1-ol benzoate and 2-methyl-2-phenylethyl propanoic acid showing the greatest effects. These findings clarify aroma-taste interactions in innovative black tea and provide a reference for sweetness modulation in tea-based foods.

## Highlights

• 93 kinds of aromatic compounds were identified that possessed sweet odor or related aromatic characteristics.
• 6 kinds of compounds were selected to reveal the aromatic compounds sweetening effect on taste flavor: *β*-bourbonene, 1-hexanol, (*Z*)-3-hexen-1-ol benzoate, 4-methoxycinnamaldehyde, 2-methyl-2-phenylethyl ester propanoic acid and citronellal.
• Molecular docking was applied to analyse the combination complexs and molecular dynamic simulation was applied to reveal the complexs stability between aromatic compounds with sweet receptors T1R3.
• The aroma additional test showed that five kinds of compound had certain improvement of sweetness in Jinmudan black tea taste.

## 1 Introduction

Tea is the second world-wild consumed beverage after water (Harfmann, 2021). Among the six traditional tea categories, black tea is characterized by a sweet fragrance and a fresh and mellow taste (Yang et al., 2020). Differed from traditional Congou black tea, innovative black teas often use aroma-enriched cultivars such *Camellia sinensis* cv. Jinmudan, Mingke No. 1, and Meizhan, and incorporate a turning-over step derived from the oolong tea manufacturing process (Wu et al., 2024). Owning to these two main factors, innovative black tea possesses pronounced floral and fruity notes in addition to sweetness (Pan, An, et al., 2025; Pan, Jia, et al., 2025). Previous studies have shown that innovative Jinmudan black tea show an excellent peach-like aroma, in which linalool (floral), phenylacetaldehyde (floral), and *δ*-decalatone (peach-like) were identified as key aroma-active compounds (Wu et al., 2023). Furthermore, the turning-over process hasbeen demonstrated to promote the accumulation of fatty acid-derived volatiles that largely contribute to the floral and fruity aroma of innovative Jinmudan black tea (Zhou, Wu, et al., 2024; Zhou, Zhu, et al., 2024).

From the perspective of flavor perception, sensory evaluation of foods mainly depends on smell and taste (Pan, An, et al., 2025; Pan, Jia, et al., 2025; Sultana et al., 2025; Zhang et al., 2025). However, most studies have focused on either odor or taste alone, ignoring their interactions. In fact, odor and taste are integrated in the brain to generate cross-modal odor-taste interactions (Charlotte et al., 2020; Halabi et al., 2021). Sweet taste is mediated by the heterodimer sweet taste receptor T1R2/T1R3, which can be activated by sucrose or other sweet ligands transduce signals to the brain (Belloir et al., 2025; Shi et al., 2025). The T1R2 subunit contains a binding pocket that can accommodate different sweeteners such as sucralose or aspartame, supporting a flexible recognition mechanism, whereas T1R3 subunit plays an essential role in receptor assembly and stabilization (Juen et al., 2025). These structural features may partly explain why individual differences in sweetness perception. Based on this concept, several studies explored odor-induced sweetness enhancement (OISE). For example, Zhou et al. found that the characteristic aroma compounds in sweet orange oil had a significant sweetening effect in sucrose solution, with limonene, citronellal, geraniol, *β*-sinensal and *β*-caryophyllene showing particularly strong effects under molecular docking analysis (Zhou, Wu, et al., 2024; Zhou, Zhu, et al., 2024). Similarly, Dai et al. demonstrated that ethyl acetate, ethyl propionate, and five other compounds from watermelon juice enhanced the sweetness of fructose solution, and identified T1R2 was the main receptor involved in OISE) (Dai et al., 2025). However, not all volatiles enhance sweetness. Cinnamyl alcohol, for instance, has been shown to neither elicit sweetness nor to enhance, and even to inhibit, the sweetness of sucralose *in vitro* and *in vivo* (Horie et al., 2025).

Cross-modal interactions between aroma and taste have also been documented in tea. Huang et al. found that nine aroma compounds in black tea induced by shaking and standing process could significantly alleviate perceived bitterness and astringency (Huang et al., 2025). Using three aroma types of Fenghuang Dancong as materials, Song et al. applied sensomics approach and identified, and jasmine lactone, (*Z*)-jasmone, linalool, *γ*-hexalactone as key aroma markers; threshold measurements in caffeine solution further indicated that caffeine lower the odor thresholds of these compounds, suggesting that caffeine can enhance of sweet, floral and fruity notes in tea infusions (Song et al., 2025). Seven volatile compounds were found to be responsible for eliciting the pleasant sensation of white tea (Baimudan) based on GC–MS, and together with molecular simulations, the neural mechanisms underlying the enhancement of sensory pleasure by tea aroma was investigated (Weiwei et al., 2025). Moreover, interactions among tea aroma compounds themselves have been explored. Zhu et al. used Longjin Tea as a model and selected (+)-(1*R*,2*S*)-methyl epijasmonate and *β*-Ionone as a case study. S-curve analysis, molecular docking, and molecular dynamics simulation revealed that hydrophobic interactions drive the formation of a stable ternary complex between these two compounds and the olfactory receptor OR52D1 (Zhu et al., 2025).

Molecular docking is a powerful technique for predicting interaction patterns, binding sites, and key forces between molecules. It can guide experimental design, reduce blind trials, and save time and costs by narrowing down candidates. This approach has been widely used in environmental monitoring (Ray et al., 2025; Steffy et al., 2025), drug discovery and design (Bhat et al., 2024; Anderson et al., 2024), medical research (Anderson et al., 2024; Halayal et al., 2024). In the food field, molecular docking has been employed to elucidate interaction mechanisms between flavor compounds and protein matrices (Jin & Wei, 2023). For example, Sun et al. combined multispectral spectroscopy, molecular docking and electronic nose analysis to investigate the interaction mechanisms between sulfur-containing flavor compounds and pea protein isolate (PPI), which could considerably alter the odor characteristics of PPI (Sun et al., 2025). Wenhua T had applied a series of spectroscopy and molecular docking techniques to find out the interaction mechanism between pyrazine and lysozyme for the purpose of understanding the possible effect of pyrazine on the structure and conformation of endogenous lysozyme. Results showed that the key mechanism was induced by static quenching for pyrazine flavor substances-Lyz interactions (Wenhua et al., 2023). Wang C had aimed at the selecting the fermented sea bass through the approaches of peptidomics, machine learning, molecular docking, results displayed the assessment of taste presentation mechanism of umami peptides by molecular docking of T1R2/T1R3, and identified umami peptides from fermented sea bass that originated from 28 precursor proteins (Wang et al., 2024).

**Fig. 1:**
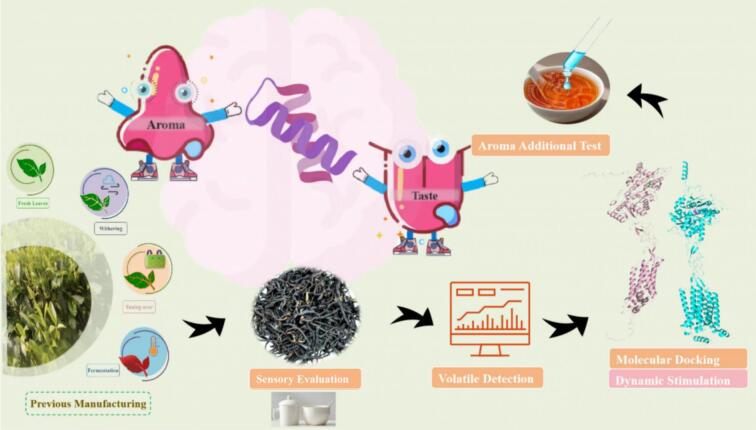
Interaction of Jinmudan black tea aroma sweetening effect in taste.

Overall, the congruence between aroma and taste attributes suggests that specific aroma compounds in innovative black tea may enhance perceived sweetness *via* direct interaction with sweet taste receptors. Nonetheless, the underlying molecular mechanisms, particularly the potential binding preference of sweet-related tea volatiles for T1R2 or T1R3, are still unclear. Therefore, in this study, innovative Jinmudan black tea was selected as the research material. Volatile profiling combined with multivariate analysis was first conducted to screen sweet aroma-associated compounds. Molecular docking and molecular dynamics simulations were then employe to explore their interactions with the sweet receptor T1R2/T1R3 (Fig. 1), and human sensory evaluation was performed to verify the sweetness-enhancing effects. This study provides new insights into aroma-taste synergy in innovative black tea and offers a theoretical basis for sweetness modulation in tea-based products.

## 2 Materials and methods

### 2.1 Tea samples collecting

A total of five Jinmudan black tea samples were collected in 2023 from Fu'an City, Fujian, China, and all were manufactured from *Camellia sinensis* cv. Jinmudan. The samples were stored at 4 °C in sealed containersuntil further analysis.

### 2.2 Sensory evaluation of Jinmudan black tea

The aroma and taste quality of Jinmudan black tea samples were evaluated by six trained tea tasters (three males and three females). All panelists had achieved professional training and had prior experience in tea sensory analysis. Evaluations were conducted in a clean and bright room with temperature at 24 °C. Tea liquor was prepared by brewing 3.0 g of black tea with 150 mL of boiled water for 5 min. Panelists assessed the overall quality, with particular emphasis on sweet-like aroma and sweet taste, and recorded aroma descriptors and taste characteristics in detail. All participants provided informed consent before participating in the study. The anonymity and confidentiality of the participants were guaranteed, and participation was completely voluntary. This test was approved by Institutional Review Board Committee of Fujian Agriculture and Forestry University.

### 2.3 Gas chromatography mass spectrometry (GC–MS) analysis

#### 2.3.1 Samples preparation

Processed tea samples were weighed, and immediately frozen in liquid nitrogen, then immersed in dry grinding cans with liquid nitrogen for 1–2 min until there were no bubbles. Each sample was loaded into clean grinding cans with steel balls, then the lids of the grinding cans were tightened, conveying grounding at the rate of 30 HZ for 30 s. Finally, the grounded tea powder was harvested and loaded into the corresponding EP tube. A total of 500 mg of tea powder was transferred immediately to a 20 mL headspace vial (Agilent, Palo Alto, CA, USA), and 3 mL saturated NaCl solution was added to inhibit potential enzymatic reactions. Vials were sealed using crimp-top caps with TFE‑silicone headspace septa (Agilent).For HS-SPME extraction, each vial was equilibrated at 60 °C for 5 min, and then a 120 μm DVB/CWR/PDMS fiber (Agilent) was exposed to the headspace for 15 min at 60 °C (Yanping et al., 2023).

#### 2.3.2 GC–MS conditions

After extraction, the fiber coating was thermally desorbed in the injection port of an Agilent 8890 GC system at 250 °C for 5 min in the splitless mode. Volatle compounds were separated on a DB-5MS column (30 m × 0.25 mm × 0.25 μm, 5 % phenyl-polymethylsiloxane, Agilent) coupled to an Agilent 7000D mass spectrometer. Helium was used as the carrier gas at a constant flow rate of 1.2 mL/min. The injector temperature was kept at 250 °C, and the detector was kept at 280 °C. The oven temperature was programmed from 40 °C (3.5 min), with the increasing rate of 10 °C/min climbed to 100 °C, then with the rate of 7 °C/min increased to 180 °C, with the rate of 25 °C/min increased to 280 °C, and held for 5 min. The mass spectra were recorded using an electron impact (EI) ionization mode at 70 eV. The quadrupole mass detector was set at the temperature of 150 °C, ion source was set at the temperature of 230 °C and transfer line temperatures were set at temperature of 280 °C, respectively. For MS, the ion monitoring (SIM) mode was used for qualitative and quantitative analysis. The characteristic fragment ions and retention times of target compounds were incorporated into the acquisition method to improve sensitivity. Retention indices (Ris) were calculated for all detected compounds, candidates were screened within an RI window defined as (RI_target_ ± 60) according to a reference RI library (Roberto et al., 2021).

### 2.4 Molecular docking analysis

Molecular docking was used to predict the binding modes between selected aroma compounds (ligands) and sweet taste receptor proteins (T1R2 and T1R3). The three-dimensional structures of the selected aromatic compounds were downloaded from the Pubchem database, and the receptor structures were downloaded from the PDB database (http://www.rcsb.org/). Docking studies were carried out using Autodock (ver 1.2.5). a Prior to docking, receptor proteins were preprocessed by removing crystallographic water molecules as appropriate, adding hydrogen atoms, and assigning charges. Putative binding sites were defined around the predicted docking pockets. Ligand structures were energy-minimized and prepared by adding hydrogens, assigning charges, and defining rotational bonds. Semi-flexible docking was employed, in which the receptor was kept rigid and the ligands were allowed limited conformational flexibility, to balance computer efficiency and accuracy. Default software parameters were used unless otherwise specified, with only the grid box parameters adjusted to cover the binding pocket. After docking, the resulting poses were ranked based on binding affinity scores, and the optimal conformations were selected for further analysis (Zhu et al., 2024).

The top six ligand-receptor complexes with the lowest (most negative) binding affinities from docking were subjected to molecular dynamics (MD) simulations using GROMACS (ver 2023). The CHARMM36 force field was applied to all protein-ligand complexes. Each complex was solvated in aTIP3P water box with appropriate dimensions, and counterions (Na^+^) were added to neutralize the system. Energy minimization was performed using the steepest descent algorithm for up to 50,000 steps to remove steric clashes. Long-range electrostatic interactions were treated using the particle mesh Ewald (PME) method, and cut-off distances for Coulombic and van der Waals interactions were set according to the standard CHARMM36 parameters. The systems were subsequently equilibrated under a constant number-volume-temperature (NVT) ensemble followed by a constant number-pressure-temperature (NPT) ensemble. Finally, production MD simulations were run for 50 ns at constant temperature and pressure. Trajectory analysis was performed using root mean square deviation (RMSD), root mean square fluctuation (RMSF), radius of gyration (Rg), ligand hydrogen bond parameters, and solvent accessible surface area (SASA). The results were displayed in graphical form using XMGRACE software (ver 5.1.25) (Andrea et al., 2022).

### 2.5 Aroma addition test and sensory evaluation

To further verify the contribution of 1-hexanol, (*Z*)-3-hexen-1-ol benzoate, 4-methoxycinnamaldehyde, 2-methyl-2-phenylethyl ester propanoic acid, and citronella to sweetness perception, an aroma addition test was conducted. Among them, 1-hexanol, (Z)-3-hexen-1-ol benzoate, 2-methyl-2-phenylethyl ester propanoic acid and citronella were all the compounds were purchased from Aladdin Ltd.(Shanghai, China)., and 4-methoxycinnamaldehyde was purchased from Adams Ltd.(Shanghai, China). The purity of these compounds was up to 98 % and all the compounds are safe. The dosage of these five compounds were set according to their average content in Jinmudan black tea samples(Table S1) and before the test, all the compounds were were diluted into pure water in advance with the ratio of 1:10 (*v*/*v*). For the aroma addition test, a Jinmudan black tea sample with weak sweet aroma and taste was selected as the base tea, and pure water in fusion of the same tea without added compounds served as the control. Tea infusions were prepared by brewing 3.0 g of tea tin 150 mL of boiling water for 5 min, followed by the addition of 0.5 mL of the diluted aroma solution. Five professional tea tasters evaluated the aroma and taste of the infusions. Sweet aroma and sweet taste intensities were scored on a 0–10 scale, where 0 represented no sweetness, 5 moderate sweetness, and 10 maximum sweetness. After each evaluation, tasters expectorated the tea and rinsed their mouths with warm water before proceeding to the next sample. All participants provided informed consent before participating in the study. The anonymity and confidentiality of the participants were ensured. The participation was voluntary and the sensory protocol was approved by the Institutional Review Board Committee of Fujian Agriculture and Forestry University.

### 2.6 Statistical analysis

Partial least squares discriminant analysis (PLS-DA) was performed using SIMCA 14 software, and variables with a VIP value (Variable Importance in Prediction) higher than 1 were considered important contributors to sample discrimination.

## 3 Results

### 3.1 Primary sensory evaluation results of Jinmudan Black Tea

**Fig. 2:**
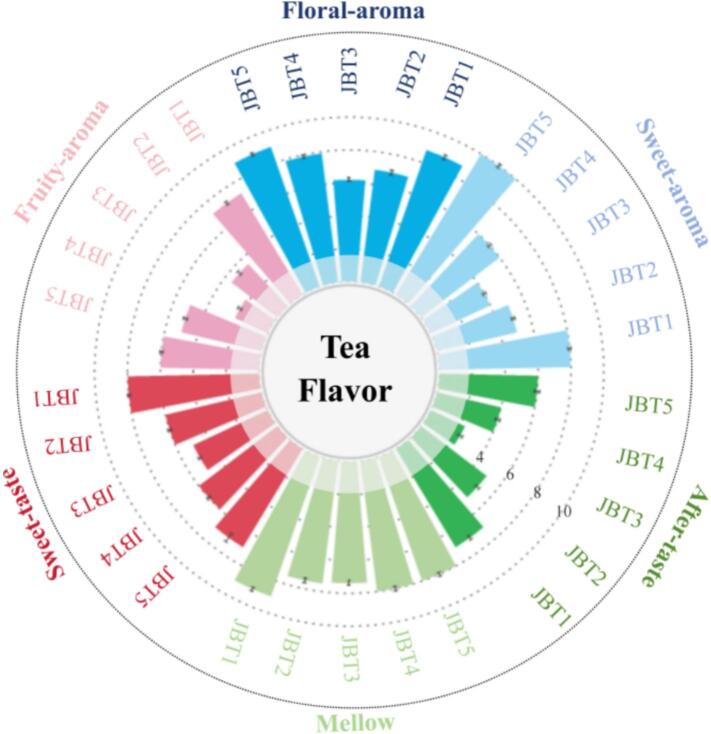
The sensory evaluation results of Jinmudan black teas.

To obtain a preliminary understanding of the quality characteristics of Jinmudan black tea, sensory evaluation was performed. The fragrance characters like ‘sweet’, ‘floral’, and ‘fruity’ as well as the taste characters like ‘sweet’, ‘mellow’, and ‘after-taste’ were picked up and measured by intensity score ranged from ‘0–10’, with ‘0∼2 showed weak, ‘3∼5’ showed moderate, ‘6∼8’ showed strong, and ‘9–10′ showed extremely strong. As shown in Fig. 2, all Jinmudan black tea samples (JBT1 ∼ JBT5) exhibited pronounced floral aroma, followed by sweet and then fruity notes, with JBT1 and JBT5 showing particularly good overall performance in both aroma and taste. For sweet taste, JBT1 received the highest score whereas JBT3 had the lowest. A similar trend was observed for after-taste, while the mellow attribute showed relatively small differences among samples. In summary, the five tea samples displayed distinct quality profiles, and JBT3 was selected for subsequent aroma addition test due to its lower sweetness-related scores.

### 3.2 The profile of sweet-related aroma compounds in Jinmudan black tea

**Fig. 3:**
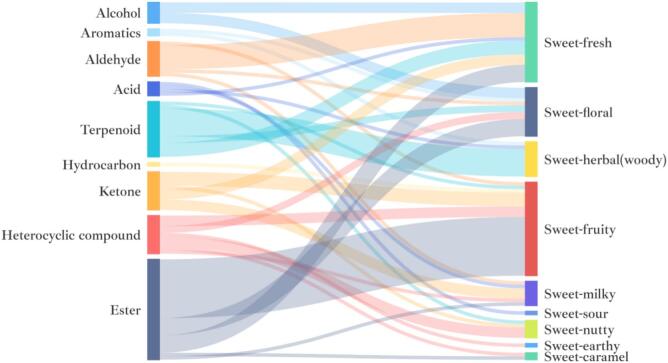
The classification of compound categories based on odor characters.

The volatile compounds in Jinmudan black tea were qualitatively and quantitatively analyzed by GC–MS. Based on flavoromics-guided selection, 48 compounds with ‘sweet’ odor, 26 with ‘fruity’ odor, and 19 with ‘floral’ odor were identified, giving a total of 93 sweet-related aroma compounds. According to the structural characteristic, these compounds could be divided into nine categories: alcohols, aromatics, aldehydes, acids, terpenoids, hydrocarbons, ketones, heterocyclic compounds, and esters. Esters accounted for the largest proportion (28 compounds), followed by terpenoids (16 compounds) while ketones and heterocyclic compounds each included 11 compounds. On the basis of odor description, these compounds were further grouped into sweet-like subtypes, namely, ‘merely sweet’, ‘sweet-floral-like’(smooth blooming-flower sweetness), ‘sweet-fruity-like’(ripe-fruit sweetness with tangy notes), ‘sweet-milky-like’ (creamy, velvety sweetness), ‘sweet-caramel-like’(toasty, caramelized sweetness), ‘sweet-nutty-like’(roasted or toasted nut-like sweetness), ‘sweet-earthy-like’(subtle, moist sweetness), ‘sweet-herbal /woody -like’(green-woody sweetness), ‘sweet-sour-like’(balanced sweet and sour profile), and ‘sweet-fresh-like’(clean, light sweetness) (Fig. 3). Most compounds fell into sweet-fruity-like (27 compounds) and sweet-fresh-like (23 compounds) groups, followed by sweet-floral-like (14 compounds) and sweet-herbal-like (10 compounds). Notably, 17 ester compounds contributed to the sweet-fruity-like fragrance, indicating that esters may play a major role in the sweet aroma and potentially in sweetness enhancement of the tea infusion. Terpenoids also showed a certain proportion in Jinmudan sweet aroma character, which mostly contributed to sweet-herbal (woody) notes. The sweet-fresh character was mostly constructed by aldehydes, imparting a pleasant and light impression. Sweet-floral odor was largely derived from esters and alcohols. In contrast, compounds responsible for sweet-milky, sweet-nutty, and sweet-caramel-like notes were fewer and may be considered as secondary contributors to the overall sweet aroma profile.

### 3.3 Selection of sweet aroma-active marker compounds in Jinmudan black tea

**Fig. 4:**
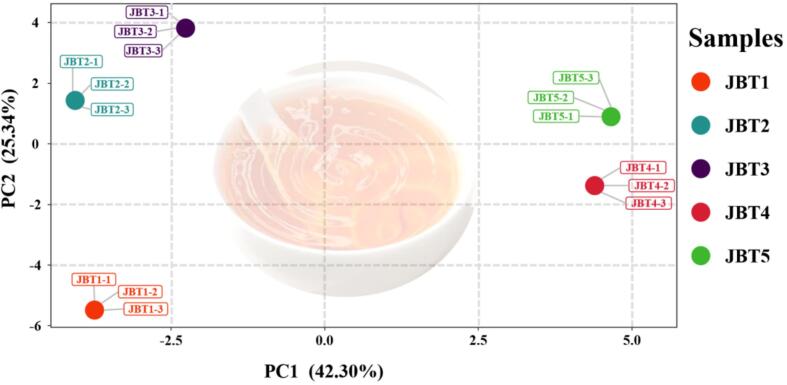
The PLS-DA analysis results of Jinmudan black teas.

To further identify sweet aroma-active marker compounds in Jinmudan black tea, PLS-DA was performed. As shown in Fig. 4, PC1 (42.30 %) and PC2 (25.34 %) together explained 67.64 % of the total variance. The coefficient of determination for Y (R^2^Y) was 0.9664 and the cross-validated R^2^ (Q^2^) was 0.9046, indicating that the model was both reliable and predictable. The five Jinmudan black tea samples could be clearly divided, and the tight clustering of replicates supported the robustness of the model. Using a VIP threshold >1, a total of 30 compounds were selected as important contributors (Table 1). Terpenoids showed generally higher VIP values, followed by esters. Among them, *β*-bourbonene had the highest value (2.052), followed by 1-hexanol (1.904), ionone (1.851), caryophyllene oxide (1.701), *α*-cedrene (1.627), (*Z*)-3-hexen-1-ol benzoate (1.571) and others, suggesting that these volatiles are potential sweet aroma-active compounds in Jinmudan black tea. Interestingly, terpenoids with high VIP values were mainly associated with sweet-herbal odor, which is consistent with sensory evaluation indicating that Jimudan black tea exhibits a pleasant green, herbal notes (Wu et al., 2023).

**Table 1:** The selected compounds under PLS-DA.

Compounds	CAS	Class	VIP	Aroma-type
*β*-Bourbonene	5208-59-3	Terpenoids	2.052	Sweet-herbal
1-Hexanol	111-27-3	Alcohol	1.904	Sweet-fruity
Ionone	8013-90-9	Terpenoids	1.851	Sweet-herbal
Caryophyllene oxide	1139-30-6	Terpenoids	1.701	Sweet-herbal
*α*-Cedrene	469-61-4	Terpenoids	1.627	Sweet-herbal
(*Z*)-3-Hexen-1-ol benzoate	25,152-85-6	Ester	1.571	Sweet-fresh
4-Methoxycinnamaldehyde	1963-36-6	Aldehyde	1.537	Sweet-milky
1-Ethenyl-1,5-dimethyl-4-hexenyl ester-pentanoic acid	10,471-96-2	Ester	1.535	Sweet-fruity
Longifolene	475-20-7	Terpenoids	1.506	Sweet-herbal
1,6,10-Dodecatrien-3-ol, 3,7,11-trimethyl-	7212-44-4	Terpenoids	1.484	Sweet-floral
Nonanoic acid methyl ester	1731-84-6	Ester	1.404	Sweet-fruity
Geranyl isobutyrate	2345-26-8	Ester	1.402	Sweet-fruity
2-Butyl-2-octenal	13,019-16-4	Alcohol	1.374	Sweet-fruity
(*E*,*E*)-2,6-Dimethyl-2,4,6-octatriene	3016-19-1	Terpenoids	1.357	Sweet-fresh
2-Methylbutyl ester-3-methyl-butanoic acid	2445-77-4	Ester	1.350	Sweet-fresh
4-(4-Methyl-3-pentenyl)-3-cyclohexene-1-carboxaldehyde	37,677-14-8	Aldehyde	1.319	Sweet-fresh
Linalyl acetate	115-95-7	Terpenoids	1.314	Sweet-herbal
1-Octen-3-ol	3391-86-4	Alcohol	1.308	Sweet-fruity
(*E*)-6,10-Dimethyl-5,9-undecadien-2-one	3796-70-1	Ketone	1.294	Sweet-fruity
(*E*)-3-Hexen-1-ol acetate	3681-82-1	Ester	1.286	Sweet-fruity
Pentanoic acid phenylmethyl ester	10,361-39-4	Ester	1.253	Sweet-fruity
4-Hydroxy-Acetophenone	99-93-4	Ketone	1.250	Sweet-milky
Citronellal	106-23-0	Terpenoids	1.239	Sweet-nutty
4-Methoxy-BenzAldehyde	123-11-5	Aldehyde	1.237	Sweet-floral
(*E*)-2-Hexenal	6728-26-3	Aldehyde	1.236	Sweet-fresh
2-Methyl-2-phenylethyl ester Propanoic acid	103-48-0	Ester	1.188	Sweet-floral
2-Methyl-2-phenylethyl ester butanoic acid	24,817-51-4	Ester	1.173	Sweet-floral
Cyclohexanecarboxylic acid methyl ester	4630-82-4	Ester	1.143	Sweet-milky
2-Propenyl ester cyclohexanepropanoic acid	2705-87-5	Ester	1.087	Sweet-fruity
2H-Pyran-3-ol,6-ethenyltetrahydro-2,2,6-trimethyl-	14,049-11-7	Heterocycles	1.014	Sweet-floral

**Fig. 5:**
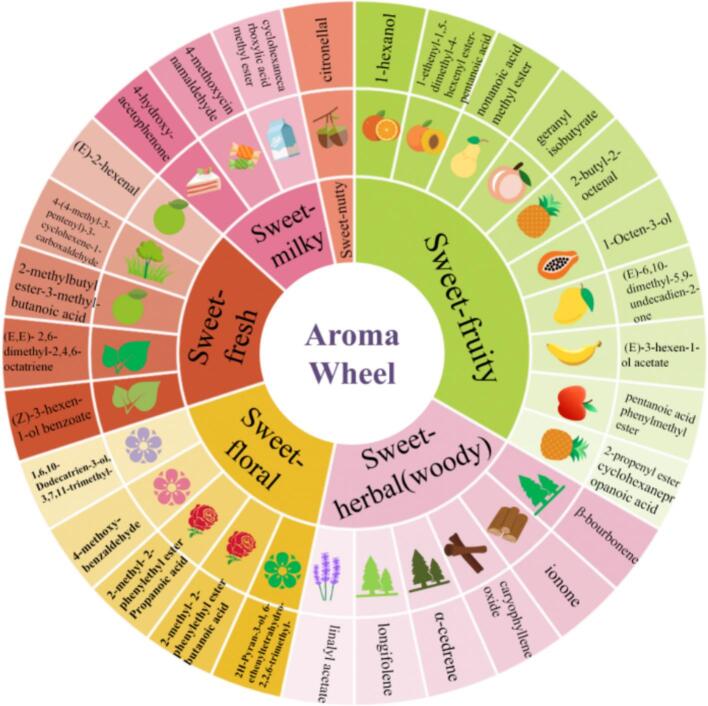
The aroma wheel of selected aroma compounds.

To provide a more intuitive overview of the odor characteristics of the 30 selected compounds, a flavor wheel was constructed based on flavoromics (Fig. 5). Different colors and sectors represented specific odor descriptors. Combined with the VIP value, in the sweet-herbal (woody) categories, *β*-bourbonene and ionone were associated with gentle, sweet woody notes. In sweet-fruity category, 1-hexanol and (*E*)-3-hexen-1-ol acetate were characterized by lively tropical fruit-like aromas. Within the sweet-fresh group, (*Z*)-3-hexen-1-ol benzoate contributed green,leafy notes. Similarly, in sweet-milky category, 4-methoxycinnamaldehyde presented a creamy, candy-like odor, while cyclohexanecarboxylic acid methyl ester imparted a smooth milky aroma. In sweet-floral category, both 2-methyl-2-phenylethyl ester propanoic acid and 2-methyl-2-phenylethyl ester butanoic acid showed elegant rose-like fragrance. Citronellal was the only compound classified in the sweet-nutty category, exhibiting a waxy, nut-like aroma.

To represent different dimensions of sweet aroma subtypes and construct a complete sweet aroma profile for Jinmudan black tea, six representative compounds were selected based on their VIP values and odor categories: *β*-bourbonene (sweet herbal/woody), 1-hexanol (sweet-fruity), (*Z*)-3-hexen-1-ol benzoate (sweet-fresh), 4-methoxycinnamaldehyde (sweet-milky), 2-methyl-2-phenylethyl ester propanoic acid (sweet-floral) and citronellal (sweet-nutty). These compounds were considered potential aromatic marker compounds for subsequent analyses. The cover the major sweet aroma subtypes identified and thus can comprehensively reflect the core component of the sweet aroma flavor of Jinmudan black tea.

### 3.4 Effect of aromatic marker compounds on sweetness in Jinmudan black tea based on molecular docking

Molecular docking was used to predict the interactions between the selected aromatic ligands and sweet taste receptors and to obtain their preferred binding conformations. In this study, six representative sweet aroma-active compounds were subjected to docking with T1R2 and T1R3. The binding affinities of these compounds with T1R2 ranged from −7.22 to −4.11 kcal/mol, and with T1R3 from −8.03 to −3.91 kcal/mol (Table 2). The differences can be attributed to the structural differences of the receptors, the physicochemical properties of the ligands, and the degree of geometric and energetic complementarity between them. Even for receptor subunits with high homology, the different intrinsic structures of subtypes and key amino acid residues in the active pocket can lead to variances in the type and strength of interaction bonds formed with the same compound. At the same time, as for the comparing between compounds, the differences in molecular structure, size, physicochemical properties, and the total energy generated by interactions with receptors, as well as the matching way, can directly lead to energy differences.

**Table 2:** Docking affinities of six aromatic compounds with T1R2 and T1R3 receptors.

Compounds	Affinity with T1R2 (kcal/mol)	Affinity with T1R3 (kcal/mol)
*β*-bourbonene	−7.22	−8.03
1-hexanol	−4.11	−3.91
(*Z*)-3-hexen-1-ol benzoate	−7.11	−6.54
4-methoxycinnamaldehyde	−6.05	−5.94
2-methyl-2-phenylethyl ester propanoic acid	−5.07	−6.22
citronellal	−4.81	−5.35

Previous studies have suggested that lower docking score indicates stronger binding affinity, with scores below −5 kcal/mol generally reflecting potential binding and scores below −7 kcal/mol indicating strong binding affinity (Li et al., 2022). Among the tested compounds, *β-*bourbonene showed the strongest binding to both T1R2 and T1R3, with docking scores of −7.22 and − 8.02 kcal/mol, respectively. In constrast, 1-hexanol exhibited the weakest affinity for both receptors, with docking scores of −4.11 and − 3.91 kcal/mol, respectively. (*Z*)-3-hexen-1-ol benzoate also displayed relatively strong binding (−7.11 and − 6.54 kcal/mol for T1R2 and T1R3, respectively), followed by 4-methoxycinnamaldehyde (−6.05 and − 5.94 kcal/mol) and 2-methyl-2-phenylethyl ester propanoic acid (−5.07 and − 6.22 kcal/mol). Citronellal showed intermediate binding, with dockingscores of −4.81 kcal/mol with T1R2 and − 5.35 kcal/mol for T1R3.

**Fig. 6:**
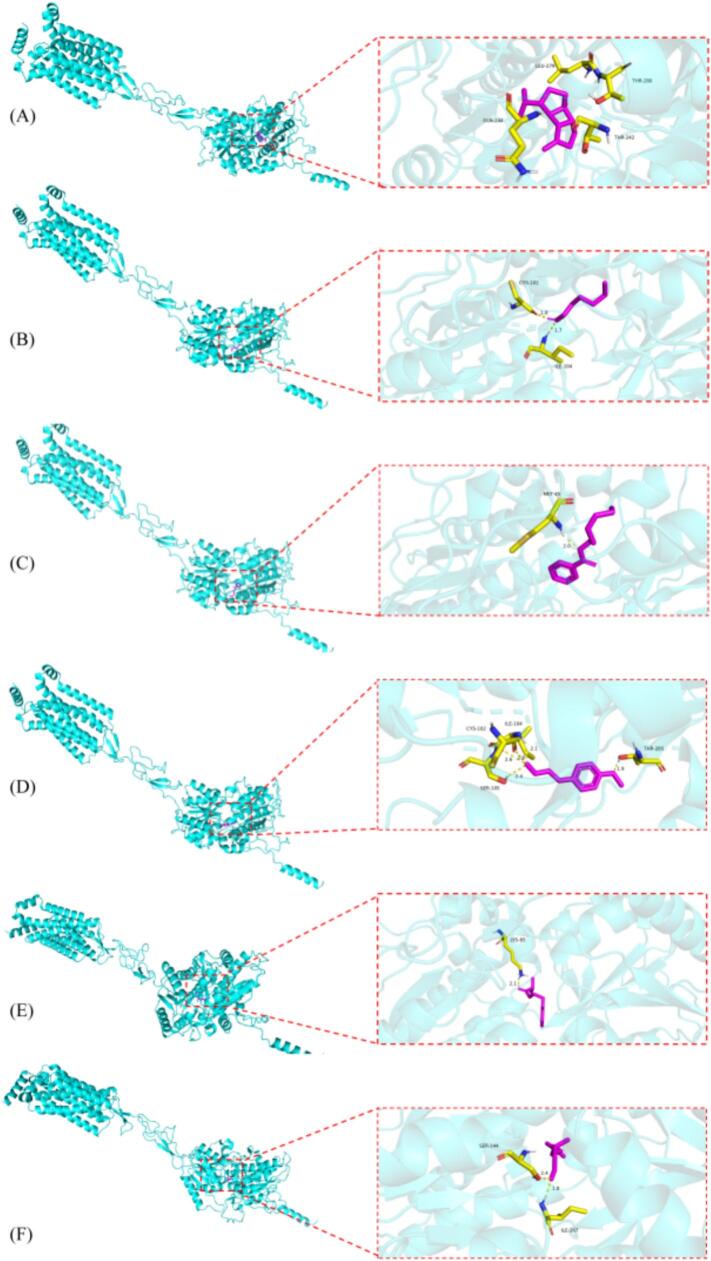
Molecular docking results of the compounds and T1R2. (A) Interaction diagram of the *β*-bourbonene-T1R2 complex; (B) Interaction diagram of the 1-hexanol-T1R2 complex; (C) Interaction diagram of the (*Z*)-3-hexen-1-ol benzoate-T1R2 complex; (D) Interaction diagram of the 4-methoxycinnamaldehyde-T1R2 complex; (E) Interaction diagram of the 2-methyl-2-phenylethyl ester propanoic acid-T1R2 complex; (F) Interaction diagram of the citronellal-T1R2 complex.

**Fig. 7:**
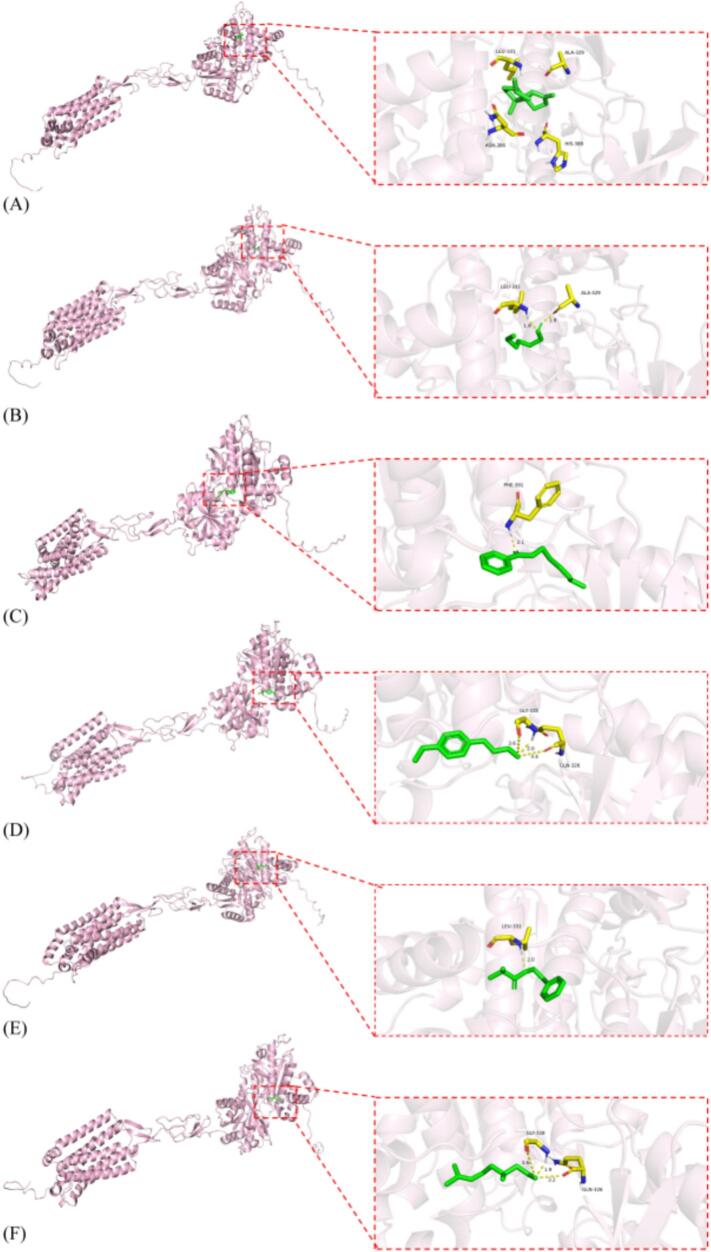
Molecular docking results of the compounds and T1R3. (A) Interaction diagram of the *β*-bourbonene-T1R3 complex; (B) Interaction diagram of the 1-hexanol-T1R3 complex; (C) Interaction diagram of the (Z)-3-hexen-1-ol benzoate-T1R3 complex; (D) Interaction diagram of the 4-methoxycinnamaldehyde-T1R3 complex; (E) Interaction diagram of the 2-methyl-2-phenylethyl ester propanoic acid-T1R3 complex; (F) Interaction diagram of the citronellal-T1R3 complex.

The binding mechanisms between the six compound targets and two receptors are shown in Fig. 6 and Fig. 7. In T1R2, there were not any hydrogen bonds connecting *β*-bourbonene and amino acid residues, speculating that other binding free energy like Van der waals force was involved to combine this compound with LEU-279, THR-280, THR-240 GLN-244; 1-hexanol formed hydrogen bonds with CYS102 (1.8 Å) and ILE104 (1.7 Å); (Z)-3-hexen-1-ol benzoate formed hydrogen bonds with MET45 (2.0 Å); 4-methoxycinnamaldehyde formed hydrogen bonds with SER105 (2.4 Å), THR (1.9 Å), CYS102 and ILE104 (2.4 Å, 2.6 Å, 2.6 Å, 2.1 Å); 2-methyl-2-phenylethyl ester propanoic acid formed hydrogen bonds with CYS (2.1 Å); citronellal formed hydrogen bonds with ILE167 (1.8 Å), SER144 (2.4 Å). In T1R3, *β*-bourbonene likely formed Van der waals force with LEU331, ALA329, ASN386, and HIS; 1-hexanol formed hydrogen bonds with ALA329 (1.9 Å), LEU331 (1.9 Å); (Z)-3-hexen-1-ol benzoate formed hydrogen bonds with PHE391 (2.1 Å); 4-methoxycinnamaldehyde formed hydrogen bonds with GLN326 and GLY328 (3.4 Å, 2.0 Å, 3.6 Å); 2-methyl-2-phenylethyl ester propanoic acid formed hydrogen bonds with LEU331 (2.0 Å); citronellal formed hydrogen bonds with GLN326 and GLY (3.2 Å, 1.9 Å, 3.6 Å). In summary, the amino acid residues ILE104, CYS102, LEU331, GLN326 and GLY328 were most likely to form hydrogen bonds with hydrogen and oxygen atoms in small molecule ligands of aromatic compounds. These amino acid residues played an important role in binding with aromatic compounds, especially GLY328 and LEU331, which have the highest chance and times respectively of forming hydrogen bonds with aromatic compounds in T1R3. This is because the GLY side chain contains carboxyl groups, while the LEU side chain contains alkyl groups, and the nitrogen atom on it will form hydrogen bonds with the oxygen atom on the aromatic compounds.

Taken together, the docking results indicate that the selected aromatic compounds generally show stronger and more favorable binding to T1R3 than to T1R2, both in terms of binding energy and interaction patterns. Therefore, the aromatic compounds-T1R3 complexes were chosen for subsequent molecular dynamics simulations to further explore the stability and dynamic behavior of these interactions.

### 3.5 Molecular dynamics simulations of the complexes

To further characterize the potential sweetening effects of Jinmudan black tea aromatic compounds at the receptor level, MD simulations were conducted for six selected T1R3 complexes: *β*-bourbonene-T1R3, 1-hexanol-T1R3, (*Z*)-3-hexen-1-ol benzoate-T1R3, 4-methoxycinnamaldehyde-T1R3, 2-methyl-2-phenylethyl ester propanoic acid-T1R3, and citronellal-T1R3. Five parameters were analyzed: root mean square deviation (RMSD), root mean square fluctuation (RMSF), protein compactness, hydrogen bond formation, and solvent accessible surface area (SASA). Binding of ligands to T1R3 may induce local or global conformational changes in the receptor, which can be captured by these descriptors.

**Fig. 8:**
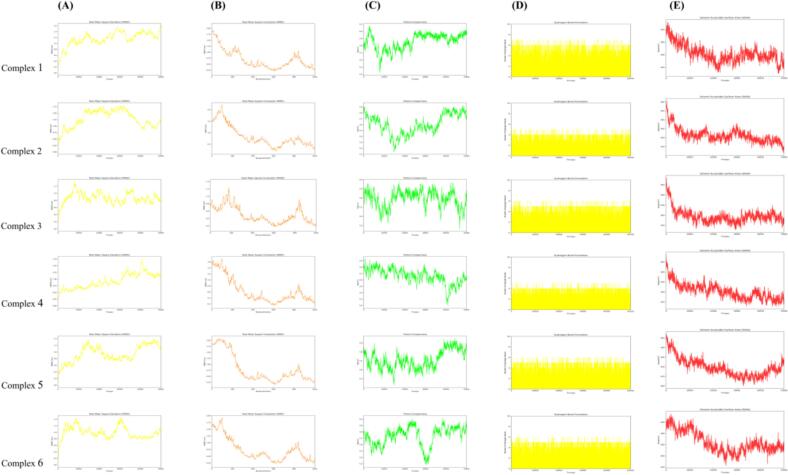
The results of molecular dynamic simulation (A) RMSD for complex1–6; (B) RMSF for complex1–6; (C) Rg for complex1 ∼ 6; (D) Hydrogen bond number for complex1 ∼ 6; (E) SASA for complex1 ∼ 6; Complex 1 ∼ 6 refers to ligand-protein in order: *β*-bourbonene-T1R3, 1-hexanol-T1R3, (*Z*)-3-hexen-1-ol benzoate-T1R3, 4-methoxycinnamaldehyde-T1R3, 2-methyl-2-phenylethyl ester propanoic acid-T1R3 and citronellal-T1R3.

RMSD was used to evaluate the overall stability of each protein–ligand complex. The RMSD trajectories reflect the time-dependent structural deviations, flexibility, and possible conformational adjustments of the complexes. As shown in Fig. 8A, all complexes exhibited initial fluctuations upon ligand binding to T1R3 and then gradually approached relatively stable trajectories. The *β*-bourbonene–T1R3 and 1-hexanol–T1R3 complexes showed more pronounced early fluctuations, whereas (*Z*)-3-hexen-1-ol benzoate–T1R3 reached its maximum deviation at approximately 10,000 ps. The 4-methoxycinnamaldehyde–T1R3 complex displayed a peak around 40,000 ps, and the 2-methyl-2-phenylethyl propanoic acid ester–T1R3 and citronellal–T1R3 complexes showed a pattern of increase–decrease–re-equilibration, indicating gradual adaptation of the receptor structure to ligand binding.

RMSF was applied to describe residue-level flexibility and to identify regions with high mobility during the simulation. As shown in Fig. 8B, the RMSF profiles of all complexes exhibited moderate fluctuations, with one major peak in an early segment of the sequence and another in a later segment. These peaks suggest that specific residues, such as GLY328 and LEU331, undergo relatively large positional fluctuations, consistent with their involvement in ligand binding as indicated by docking analysis.

Protein compactness, which influences stability and function, was assessed using the radius of gyration (Rg). As shown in Fig. 8C, all complexes fluctuated below an Rg value of approximately 6 nm throughout the simulations, indicating that the global protein structure remained compact. Notably, the citronellal–T1R3 complex reached an Rg minimum of about 5.3 nm at 30,000 ps, while the 4-methoxycinnamaldehyde–T1R3 complex decreased to around 5.2 nm at 40,000 ps, suggesting slightly tighter packing of the receptor in these ligand-bound states.

The number of hydrogen bonds between ligand and receptor reflects an important component of the stabilizing forces within each complex. As shown in Fig. 8D, the number of hydrogen bonds generally ranged from 4 to 7 across all complexes during the simulation. Interestingly, the *β*-bourbonene–T1R3 complex exhibited the highest average number of hydrogen bonds over time, which is not fully consistent with the docking snapshot that suggested mainly hydrophobic interactions. This discrepancy may result from different binding poses sampled during MD or from additional stabilizing interactions that emerged under dynamic conditions.

SASA was used as an indicator of protein surface exposure and folding stability. As shown in Fig. 8E, all complexes displayed a similar decrease in SASA at the early stage of the simulation, followed by convergence to relatively stable values, indicating that the protein–ligand systems reached equilibrated and compact conformations.

Overall, the combined RMSD, RMSF, Rg, hydrogen bond, and SASA analyses support the docking results by demonstrating that the selected aromatic compounds form stable complexes with T1R3, maintained by hydrophobic interactions and hydrogen bonding. These findings corroborate the proposed molecular mechanism by which sweet aroma-active compounds from Jinmudan black tea may modulate sweet taste perception through interaction with the T1R3 subunit.

### 3.6 Aroma addition test for verification of sweetening effect

A targeted aroma addition test was performed to verify the sweetening effects of the selected aromatic marker compounds. Five compounds, namely 1-hexanol, (*Z*)-3-hexen-1-ol benzoate, 2-methyl-2-phenylethyl ester propanoic acid, citronella, and 4-methoxycinnamaldehyde were tested (*β*-bourbonene was excluded because it was not commercially available). A Jinmudan black tea sample with weak sweet aroma and taste was brewed with each compound solution for 5 min, and trained tea tasters evaluated the resulting infusions for sweet aroma and sweet taste.

The results showed that the addition of (*Z*)-3-hexen-1-ol benzoate produced the best sweetening effect (7.83 ± 0.98), followed by 2-methyl-2-phenylethyl ester propanoic acid (7.00 ± 0.89). The other three compounds yielded moderate increases in sweetness. Interestingly, panelists also noted a slight reduction in perceived astringency in the samples with added aroma compounds. This phenomenon may be related to multisensory integration in the brain, where taste, smell, and even trigeminal sensations are combined to form a unified flavor experience, and where sweetness can modulate or mask bitterness and astringency (Elena et al., 2022; Wang et al., 2016).

## 4 Discussion

Sugar, artificial sweeteners, and other sweet-tasting compounds activate the human sweet taste receptor, a heterodimer composed of T1R2 and T1R3 (Zhao et al., 2003). T1R2 contains a binding pocket that can accommodate structurally diverse sweeteners such as sucralose and aspartame, indicating a flexible recognition mechanism, whereas T1R3 plays a key auxiliary role in receptor assembly and stabilization and can modulate ligand sensitivity (Juen et al., 2025). Inter-individual differences in receptor structure and function may therefore contribute to variability in sweetness perception. A better understanding of how additional chemicals enhance sweetness provides a basis for designing safer, less cloying, low-calorie sweeteners that help reduce sugar intake without compromising palatability, thereby contributing to the prevention of obesity and diabetes.

From a sensory perspective, foods with appropriate sweetness, saltiness, and umami are generally more acceptable, whereas strong bitterness tends to reduce consumer preference. Taste-active compounds can not only increase sweetness but also suppress bitterness by elevating bitterness perception thresholds or by masking bitter notes. For example, the combined use of umami substances and sweeteners significantly increased the bitterness threshold of a simulated bitter Tartary buckwheat flour solution (from 0.078 mg/mL to 0.239 mg/mL), thereby improving physical and psychological tolerance to bitterness (Chang et al., 2025). Similarly, the natural bitterness of green tea extract limits its use in food applications, but complexation with *β*-cyclodextrin has been shown to mask bitterness while preserving functional properties (Li et al., 2025). These findings highlight the importance of multisensory and cross-modal strategies for optimizing flavor.

Sweet taste is a crucial component of black tea flavor quality. Recent work has demonstrated that different tea types differ markedly in soluble sugar content and composition, largely due to differences in processing steps that regulate sugar metabolism pathways such as starch and sucrose metabolism and galactose metabolism (Li et al., 2025). Compared with pan-fired green tea and sun-withered white tea, the fermentation step in black tea processing promotes the accumulation of d-fructose and glucose; as d-fructose is approximately 1.8 times sweeter than sucrose, this shift in sugar profile can substantially enhance sweetness in black tea infusions. Innovative black teas produced by incorporating an oolong-style turning-over step have been reported to show higher quality in both aroma and taste. Contributing factors may include the use of aroma-enriched cultivars, more mature leaves with higher soluble sugar content, and processing conditions that favor the formation of key volatiles (Wu et al., 2023; Qingyang Dilpreet et al., 2024). Under such conditions, there is increased potential for interactions between taste-active sugars and aroma-active volatiles.

In this context, the present study provides molecular-level evidence that selected sweet aroma-active compounds from Jinmudan black tea can interact with the sweet receptor, particularly T1R3, and thus may contribute to odor-induced sweetness enhancement. By combining flavoromics-guided volatile profiling, multivariate analysis, molecular docking, MD simulations, and targeted sensory validation, we show that specific volatiles not only shape the sweet aroma profile but also have the potential to modulate sweet taste perception *via* receptor-level interactions. These mechanistic insights support the concept that optimizing both soluble sugars and key aroma compounds—through cultivar selection and processing design—could be an effective strategy to fine-tune sweetness and overall flavor in innovative black teas and related tea-based products.

## 5 Conclusion

This study used innovative Jinmudan black tea to link sweet-related aroma volatiles with sweetness enhancement at both receptor and sensory levels. Flavoromics-based GC–MS profiling identified 93 sweet-associated volatiles, and PLS-DA (VIP > 1) highlighted 30 key contributors. Six representative sweet aroma-active compounds covering herbal/woody, fruity, fresh, milky, floral, and nutty subtypes were selected as marker volatiles. Molecular docking and molecular dynamics simulations showed that these compounds bind more strongly and stably to the sweet taste receptor subunit T1R3 than to T1R2, mainly through hydrophobic interactions and hydrogen bonding. Aroma addition tests confirmed that all five commercially available markers increased perceived sweetness in Jinmudan black tea, with (*Z*)-3-hexen-1-ol benzoate and 2-methyl-2-phenylethyl propanoic acid ester showing the strongest effects. Overall, this work reveals a mechanistic link between specific sweet aroma-active volatiles and sweet taste perception in innovative black tea and provides a practical framework for modulating sweetness and developing sugar-reduced, yet palatable, tea-based products.

## CRediT authorship contribution statement

**Wu Yang:** Writing – original draft, Visualization. **Wu Qingyang:** Investigation, Formal analysis. **Zhou Ziwei:** Validation, Software. **Deng Tingting:** Formal analysis, Data curation. **Sun Yun:** Writing – original draft, Visualization, Formal analysis, Data curation.

## Funding

This work was supported by the National Natural Science Foundation of China Agriculture Reasearch System of MOF and MARA (CARS-19), the Natural Science Foundation of Fujian, China (2022J05271), and the Scientific Research Foundation of Ningde Normal University (2022Y05).

## Declaration of competing interest

The authors declare that they have no known competing financial interests or personal relationships that could have appeared to influence the work reported in this paper.

## Data Availability

Data will be made available on request.

## Appendix A Supplementary data

Supplementary material Image 1
