# Supplementary material for: Identification of key aromatic compounds responsible for sweetening sensory effect in Jinmudan black tea though flavoromics and molecular modeling
Source: Food Chem X. 2025 Nov 29;32:103314. doi: 10.1016/j.fochx.2025.103314 (PMC12719204; doi:10.1016/j.fochx.2025.103314)
Supplement: Supplementary file 1 — Supplementary material [file mmc1.docx]

Table S1 The average content of the selected compounds used in aroma additional adding test

| Compounds Name | Average Content(μg/g) |
| --- | --- |
| 1-hexanol | 13.8987 |
| (Z)-3-hexen-1-ol benzoate | 12.1168 |
| 2-methyl-2-phenylethyl ester propanoic acid | 7.4967 |
| citronella | 27.9458 |
| 4-methoxycinnamaldehyde | 15.7993 |
